# Outcomes of pars plana vitrectomy in three cases of ophthalmomyiasis interna

**DOI:** 10.1016/j.ajoc.2022.101697

**Published:** 2022-09-08

**Authors:** Lukpan Orazbekov, Elmira Kanafyanova, Kairat Ruslanuly

**Affiliations:** a First Ophthalmology Department, Kazakh Eye Research Institute, Almaty, Kazakhstan; b Postgraduate Education Department, Kazakh Eye Research Institute, Almaty, Kazakhstan

**Keywords:** Ophthalmomyiasis, Ocular parasites, Intraocular larva

## Abstract

**Purpose:**

To report the outcomes of pars plana vitrectomy in three cases of ophthalmomyiasis interna.

**Observations:**

Case 1 is a 15-year-old male with a mobile lenticular gray-white larva in the vitreous. Case 2 is a 4-year-old female with a floating larva in the optic axis. Case 3 is an 8-year-old female with a floating larva in the optic axis; however, intraoperatively, the larva was found subretinally. In all cases, 25-gauge pars plana vitrectomy was performed.

**Conclusions and importance:**

Ophthalmomyiasis interna is a rare parasitic eye disease that occurs when larvae of flies or gadflies enter the anterior chamber or/and posterior segment. Pars plana vitrectomy is an effective method of dealing with ophthalmomyiasis interna allowing to reach the main goal of treatment: removing the larva and restoring the eye structures. It is necessary to start treatment as soon as possible to prevent complications associated with mechanical injury and the prolonged toxic effect of larval immunogenic materials.

## 1 Introduction

Ophthalmomyiasis interna (OI) is a rare parasitic eye disease that occurs when fly or gadfly larvae enter the anterior chamber or/and posterior segment. Clinical manifestations range from accidental detection in asymptomatic courses to sudden vision loss due to severe uveitis and retinal detachment. Given the rarity and burdensome consequences of this pathology, the complexity of diagnosis, and the incomprehensibility of treatment tactics in such cases, it seemed interesting to us to report our cases of OI from the main referral ophthalmological center of Kazakhstan over the past twelve years.

## 2 Findings

Case №1. In 2009, a 15-year-old male from a rural area presented complaining of eye redness, vision loss, and severe pain in the right eye for one month. Prior to the onset of symptoms, he recalled that three months ago, when he was herding cattle, he felt something (presumably, an insect) fly into his right eye. In a local hospital, chorioretinitis was diagnosed and she was referred to the Kazakh Eye Research Institute (KazERI).

**Fig. 1:**
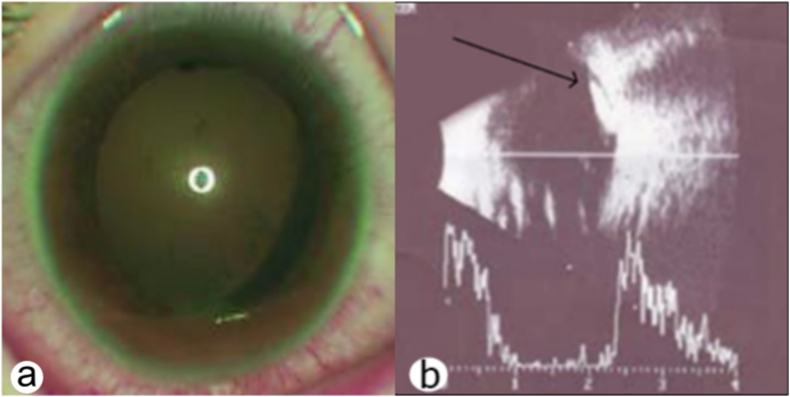
Case №1. a. Clinical presentation of internal ophthalmomyiasis. b. Ocular ultrasound demonstrating a larva (arrow).

Best-corrected Snellen visual acuity (BCVA) was 20/640 in the right eye and 20/20 in the left eye. The right eye slit-lamp examination (SLE) showed conjunctival injection, iridodonesis with posterior synechiae, and lens subluxation with vacuoles in the posterior cortex and posterior capsule opacification (PCO) (shown in Fig. 1 a). On dilated fundus examination (DFE), a mobile lenticular gray-white larva ≈ four mm in length and inflammatory cells with hemorrhagic masses were observed in the vitreous. No changes were observed in the contralateral eye. Ocular ultrasound showed a freely floating mobile larva in the vitreous (shown in Fig. 1 b). We diagnosed - OI. A topical corticosteroid (dexamethasone 0.1%) and a systemic corticosteroid (prednisolone sodium phosphate injection 30 mg/ml - 1 mg/kg/day) were administered for seven days. The surgery was scheduled for the following day.

We performed phacoaspiration with intraocular lens (IOL) implantation using a capsular tension ring. Vitreous cavity examination showed the larva on the retinal surface of the posterior pole, sub- and preretinal exudates, multiple microhemorrhages, and severe retinal edema. Then we continued surgery by performing 25-gauge pars plana vitrectomy (PPV) (The CONSTELLATION® Vision System, Alcon). Then we removed the port at 10 o'clock and extracted the larva with the Alcon 25+ Grieshaber MaxGrip DSP forceps via the trocar sclerotomy from the vitreous cavity. Subsequently, we performed retinal endolaser coagulation with injection of perfluorocarbon liquid, which was removed on the fifth day. A topical antibiotic (levofloxacin 0.5%) was administered for two weeks. The extracted larva was identified as the larva of a stable fly (Stomoxys calcitrans) covered with fibers. In other words, the larva was at the beginning of development. Postoperatively, BCVA of the right eye was 20/250.

Case №2. In 2015, a 4-year-old female from a rural area presented complaining of eye redness, vision loss, and pain in the right eye for one month. In a local hospital, acute uveitis was diagnosed and she was referred to the KazERI.

**Fig. 2:**
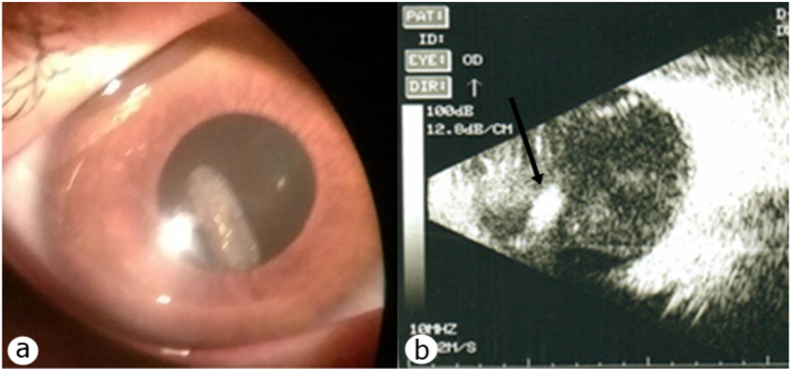
Case №2. a. On slit-lamp examination, a larva is visualized in the vitreous posteriorly to the lens capsule. b. Ocular ultrasound demonstrating a larva (black arrow).

BCVA was light perception in the right eye and 20/20 in the left eye. The right eye SLE showed conjunctival injection, PCO, floating larva in the optic axis (shown in Fig. 2 a). On DFE, a mobile lenticular gray-white larva ≈ six mm in length with inflammatory cells in the vitreous were observed. Ocular ultrasound showed a freely floating mobile larva in the vitreous (shown in Fig. 2 b). No changes were observed in the contralateral eye. We diagnosed - OI. Topical and systemic corticosteroids were administered for seven days. The surgery was scheduled for the following day.

We performed a lensectomy. Vitreous cavity examination showed the encapsulated larva was found preretinally in the macular region, preretinal exudates, and retinal edema. Then we performed 25-gauge PPV (The CONSTELLATION® Vision System, Alcon), the capsule was incised with a spatula (ALCON GRIESHABER 23G 45° DELAMINATION SPATULA). Then we removed the port at 10 o'clock and extracted the larva with the Alcon 25+ Grieshaber MaxGrip DSP forceps via the trocar sclerotomy from the vitreous cavity. A topical antibiotic (levofloxacin 0.5%) was administered for two weeks. The extracted larva was identified as the larva of a botfly (Oestrus ovis). Three months later, we implanted IOL. The final BCVA of the right eye reached 20/32.

Case №3. In 2020, an 8-year-old female from a rural area presented complaining of vision loss in the left eye for three months. Prior to the onset of symptoms, he recalled that five months ago, she noted blurred vision and eye redness in the left eye. In a local hospital, she was treated for dry eye syndrome. Two months later, besides the persistence of eye redness, she noted a loss of vision in the left eye. In a local hospital, chorioretinitis was diagnosed and she was referred to the KazERI.

**Fig. 3:**
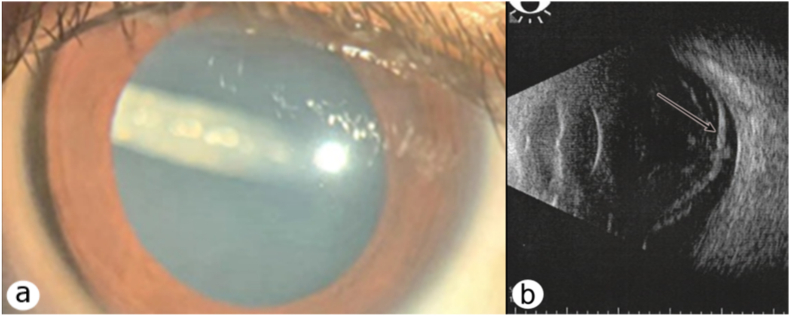
Case №3. a. On slit-lamp examination, a larva is visualized in the vitreous posteriorly to the lens capsule. b. Ocular ultrasound demonstrating retinal detachment (white-black arrow).

BCVA was 20/20 in the right eye and counting fingers at one foot in the left eye. The left eye SLE showed PCO and an actively floating larva in the optic axis (shown in Fig. 3 a). On DFE, a mobile lenticular gray-white larva ≈ eight mm in length, vitreous opacity, and a retinal detachment were observed. Ocular ultrasound showed a retinal detachment (shown in Fig. 3 b). No changes were observed in the contralateral eye. We diagnosed - OI. Topical and systemic corticosteroids were administered for seven days. The surgery was scheduled for the following day.

**Fig. 4:**
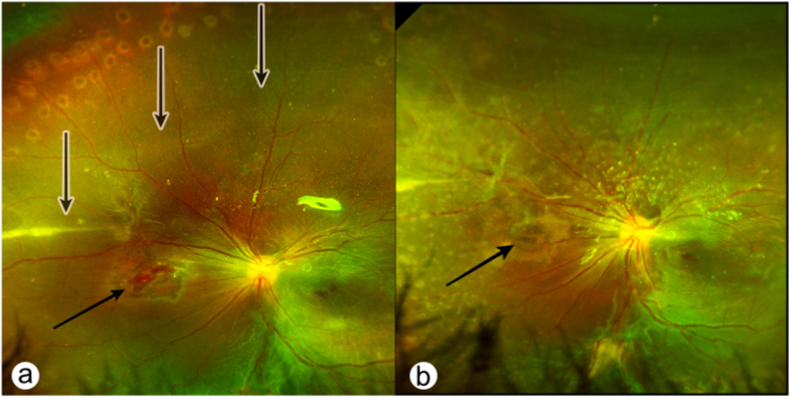
Fundus image of Case №3 immediately after surgery (a) and a month later (b). Black arrows point to the site where the larva was extracted subretinally. White-black arrows point to the intra- and subretinal migration tracks of the larva.

We started the surgery with phacoaspiration and IOL implantation. Vitreous cavity examination showed no larva but a retinal tear located nasally to the optic nerve and total rhegmatogenous retinal detachment. Then we performed 25-gauge PPV (The CONSTELLATION® Vision System, Alcon) with pneumatic retinopexy, and once the retina was flattened, the larva was visualized subretinally. We carefully moved the larva toward the tear and extracted the larva through it into the vitreous cavity. Then we removed the port at 10 o'clock and extracted the larva with the Alcon 25+ Grieshaber MaxGrip DSP forceps via the trocar sclerotomy from the vitreous cavity followed by silicone oil tamponade and endolaser photocoagulation (shown in Fig. 4). A topical antibiotic (levofloxacin 0.5%) was administered for two weeks. The extracted larva was identified as the larva of the bazaar fly or eye-seeking fly (Musca Sorbens). Postoperatively, BCVA of the right eye was 20/400.

## 3 Discussion

According to the available literature, OI occurs in all age groups, rarer in the elderly.^1^, ^2^, ^3^, ^4^, ^5^, ^6^, ^7^, ^8^, ^9^, ^10^ In our case series, all three cases of OI occurred in children from four to fifteen years of age. One of the main contributing factors responsible for OI at a younger age could be related to the psychology of children; they do not tend to clean the conjunctival sac on their own as adults do.^11^ Moreover, children tend not to talk to their parents about their injuries and events that bother them and tend to mask a decreased vision in one eye. Due to the young age of patients, the circumstances of OI are unknown, except for the first case where something flew into his eye. In the other two cases, the only thing that was known was that they resided near livestock. We did not find any ocular wounds/injuries or larval penetration sites on SLE in our case series. The patients were not treated prior to referring to KazERI, except for a patient in Case №3 who was treated for dry eye with hyaluronic acid-containing topical eye drops. All larvae were in the posterior segment of the eye globe, freely moving from the posterior lens capsule to the subretinal space, which probably explains why initially the diagnosis of OI was not made, because of the difficulty in diagnosing internal ophthalmomyiasis due to being outside the optic axis. In all cases, PCO was an indication for cataract surgery. Sub- and preretinal exudates, retinal edema, and multiple microhemorrhages were observed intraoperatively on the retinas involving the macular area. Endolaser photocoagulation was performed paravasally in the sites of retinal damage in all cases. After PPV with larval removal, we observed resolution of the inflammatory process with the restoration of ocular media transparency and visual improvement in all cases. In Case №1, inability to perform adequate retinal laser photocoagulation, we decided to perform a temporary perfluorocarbon tamponade until the formation of pigmented laser spots. Despite the small number of patients, we found a direct correlation between the time the larva was in the eye and BCVA after surgery. In Case №3, the larva had been in the eye for five months, the visual outcome was the lowest (20/400), whereas in Case №2, the larva had been in the eye for one month, the visual outcome was the highest (20/32), suggesting the importance of early diagnosis and early surgical treatment. In all cases, we used topical and systemic corticosteroids to reduce eosinophil-mediated tissue inflammatory responses, which develop as a reaction to immunogenic larval material.^12^ To prevent a secondary bacterial infection, we started topical antibiotics in all cases.

Syrdalen et al. reported a case of a 13-year-old boy from Norway; the reindeer warblefly (Oedemagena tarandi) caused a defect of the zonular apparatus, lens subluxation, microhemorrhages, and pigmented tracks.^1^ In another paper, Syrdalen and Stenkula reported 6 cases of OI and, in all cases, the reindeer warblefly (Oedemagena tarandi) was the cause.^2^ All six patients were male, four children aged 7–13 years and two adults aged 43 and 50 years. Of the six patients, two children and two adults had subluxation of the lens, while simultaneous retinal hemorrhage and retinal detachment were present in three children and one adult. Unfortunately, two children with retinal detachments eventually lost their vision due to proliferative vitreoretinopathy, which developed in the long term. In our case series, lens subluxation was observed in Case №1 and retinal detachment in Case №3.

Another contributing factor is probably the morphological changes of the sclera with age; an infant's sclera is thinner than an adult's.^13^ Myofibroblasts, contractile cells, appear in the sclera from one to four years of age, and their concentration increases with age.^14^ Evidence from another study shows that the increase of myofibroblasts with age results in forming a stronger and stiffer matrix.^15^ Thus, a low concentration of contractile cells in the pediatric scleral matrix was presumably one of the factors contributing to larval penetration in our case series.

Regarding anatomical features of the larvae in our case series, Stomoxys calcitrans is similar in appearance to Musca sorbens: both have a pale yellow to creamy white “vermiform” bodies with a single mouth hook. Additionally, Stomoxys calcitrans had anterior spine bands. Oestrus ovis is a spindle-shaped larva with two mouth hooks and abdominal intersegmental spine bands. Although the mechanism for OI in humans is not clearly understood, it is known that the larvae can attach to any mucosal surface and consume nutrients with mouth hooks and crawl freely with the help of spines.^16^ We suggest that mouth hooks are a tool of penetration of the sclera of these three larvae, since it is known that Oestrus ovis usually breeds in the nasal cavity and sometimes they are found in the sinuses.^16^

On Medline and web search, there is no guideline for the treatment and management of OI. However, there are several reports on various types of OI management.

The first approach is destroying the larva with photocoagulation, focusing laser shots at the larval head or body.^3^^,^^4^^,^^17^, ^18^, ^19^ Georgalas et al. reported a 27-year-old woman who suffered orbital cellulitis and visual impairment after a South African safari.^3^ They observed one larva in the angle of the anterior chamber and one rapid-moving larva in the subretinal space. They successfully applied argon laser photocoagulation to subretinal larva. Then they tried to wash out the larva from the anterior chamber. However, the larva disappeared from the anterior chamber. The following day, the larva was observed in the vitreous, and the argon laser photocoagulation was applied to the second larva. Buettner reported another interesting case of a 41-year-old herdsman from the USA who suffered from blurred vision and crisscrossing discolored lines in the central visual field.^4^ Cuterebra larva was observed temporal to the macula with gray-white tracks crisscrossing all fundus with several tracks converging toward a chorioretinal scar near the temporal ora serrata, presumably the site of larva penetration. In this case, the larva was also destroyed by argon laser photocoagulation. However, the drawbacks of this method are that media opacities persist, lowering existing poor BCVA, and the decomposing larva remains inside the eye, continuing to expose the retina to its immunogenic materials’ toxic effects. Based on a literature review, the following argon laser settings were used: power 350–400 mW, duration 0.1–0.2 seconds, and spot size 200 μm.^17^, ^18^, ^19^ The total number of laser spots ranged from 10 to 330 until the larva was no longer mobile.

The second approach is using oral antiparasitic drugs: there is a report of OI's successful treatment with Ivermectin.^5^ Taba et al. reported a case of successful conservative treatment of an 11-year-old boy with a five-day history of decreased visual acuity and photophobia. On the initial examination, BCVA was 20/80. DFE showed subretinal pigmentation, disc edema, congested retinal veins, and two areas of subretinal hemorrhage were observed; detailed examination failed to reveal a motile larva. Five days later, BCVA worsened to 20/200 and they decided to use a single oral dose of 200 mg/kg of ivermectin followed by oral prednisone two days later. Five days later, BCVA reached 20/60, a month later - 20/40, eight months later - 20/20. There are two points worth mentioning about this method. First, this is a single reported successful case of OI treatment. Second, the larva itself was not found with detailed examination.

The third approach is performing PPV if the larva is found in the vitreous.^1^^,^^6^ Although there is a risk of postoperative hypotony, choroidal, or retinal detachment in PPV, the advantages of PPV are immediate relief of inflammation and pain, ensuring transparency of the visual media, and rapid visual recovery. In our case series, PPV allowed us to extract the larva, preserve relatively good BCVA, and restore the intraocular structures as far as possible.

There are no previous reports of OI from our country, since there is no epidemiological data on OI to report. Moreover, there are no available reported data on OI from our neighbouring countries in Central Asia or the Commonwealth of Independent States countries, except Ukraine.^7^ A 7-year-old female patient had been suffering from intense vitreous opacity and OI for one month. Once 20-gauge PPV was performed and the larva was removed from the vitreous cavity, a retinal tear and multiple hypopigmented lines on the attached retina were found; endolaser photocoagulation was performed. The extracted larva was identified as Hypoderma bovis. The authors suggested that the larva had moved freely between the subchoroidal space and the vitreous cavity.

One more issue worth raising is the endemicity of ophthalmomyiasis. In the literature review on Medline, we encountered phrases: “ophthalmomyiasis is not endemic to our region.” However, there have been case reports on ophthalmomyiasis since the 1920s from the whole world: from the cold northern countries to the hot equatorial and tropical ones.^8^, ^9^, ^10^ For instance, the climate in Kazakhstan is severely continental with hot summers and cold winters. Therefore, we assume that the phenomenon of ophthalmomyiasis is not related to a region or climate but related merely to the presence of flies or gadflies in human habitats.

## 4 Conclusions

Early diagnosis and management of ophthalmomyiasis are important in preventing complications. There are no guidelines or systemic treatments for this condition and analysis of the effectiveness of antiparasitic drugs in treating OI. If OI is suspected, it is necessary to perform PPV as soon as possible to prevent complications associated with mechanical injury and the prolonged toxic effect of larval immunogenic materials in the eye that may result in irreversible visual impairments.

### Patient consent

Written informed consent was obtained from the legal guardians of the patients for publication of these case reports and any accompanying images.

## Funding

No funding or grant support.

## Authorship

All authors attest that they meet the current ICMJE criteria for Authorship.

## Declaration of competing interest

The authors declare that they have no known competing financial interests or personal relationships that could have appeared to influence the work reported in this paper.

## Appendix A Supplementary data

Fig. S1 Optical coherence tomography of the macula in Case №2, 7 days after surgery. Macular profile changed. Remnants of larval capsule. Fig. S1

Fig. S2 Optical coherence tomography of the macula in Case №3, 7 days after surgery. Diffuse macular edema. Fig. S2
