# Supplementary figures and images for: Outcomes of pars plana vitrectomy in three cases of ophthalmomyiasis interna
Source: Am J Ophthalmol Case Rep. 2022 Sep 8;28:101697. doi: 10.1016/j.ajoc.2022.101697 (PMC9474313; doi:10.1016/j.ajoc.2022.101697)

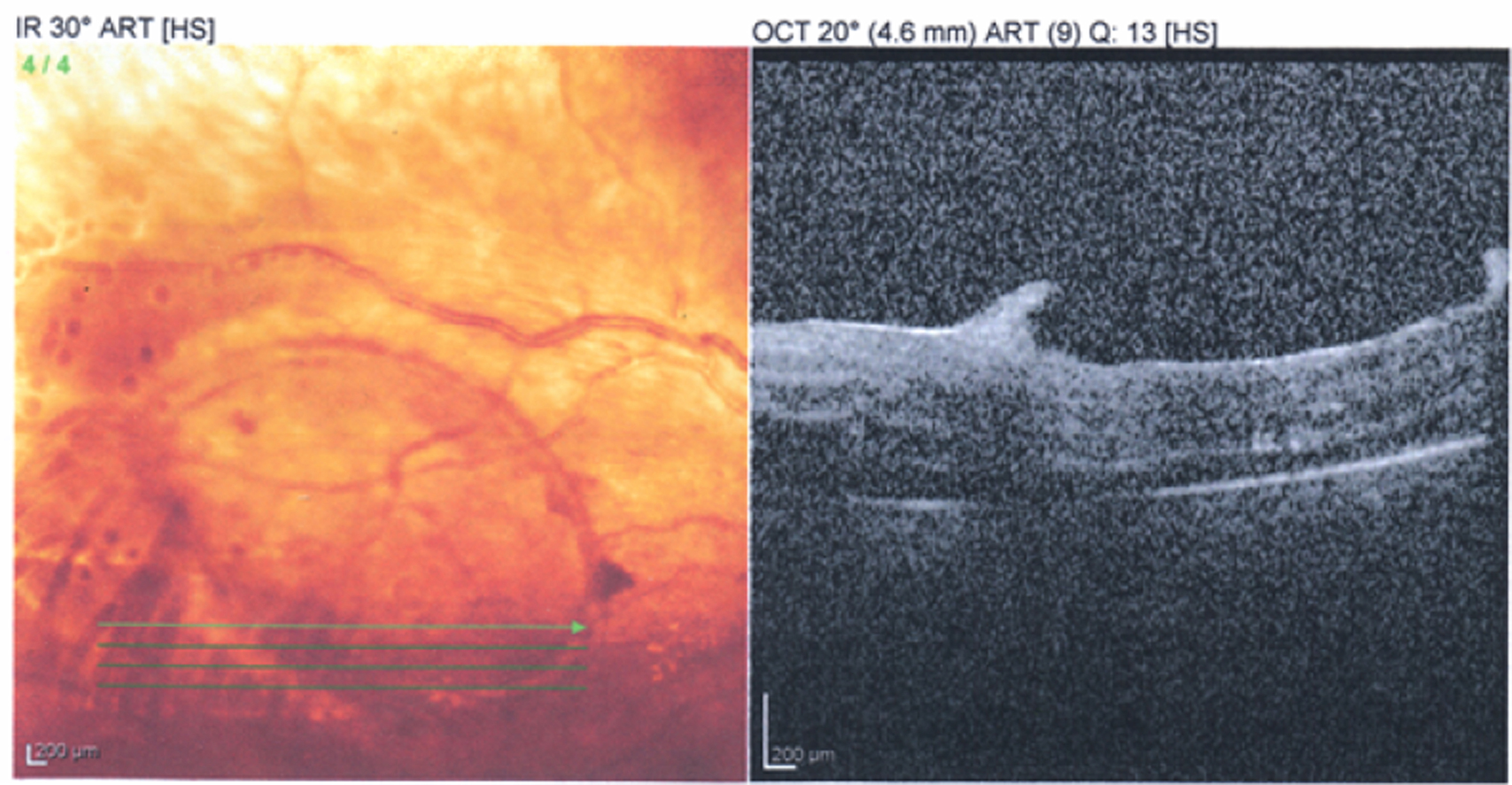

Supplement: Fig. S1 — Optical coherence tomography of the macula in Case №2, 7 days after surgery. Macular profile changed. Remnants of larval capsule. [file mmcfigs1.jpg]

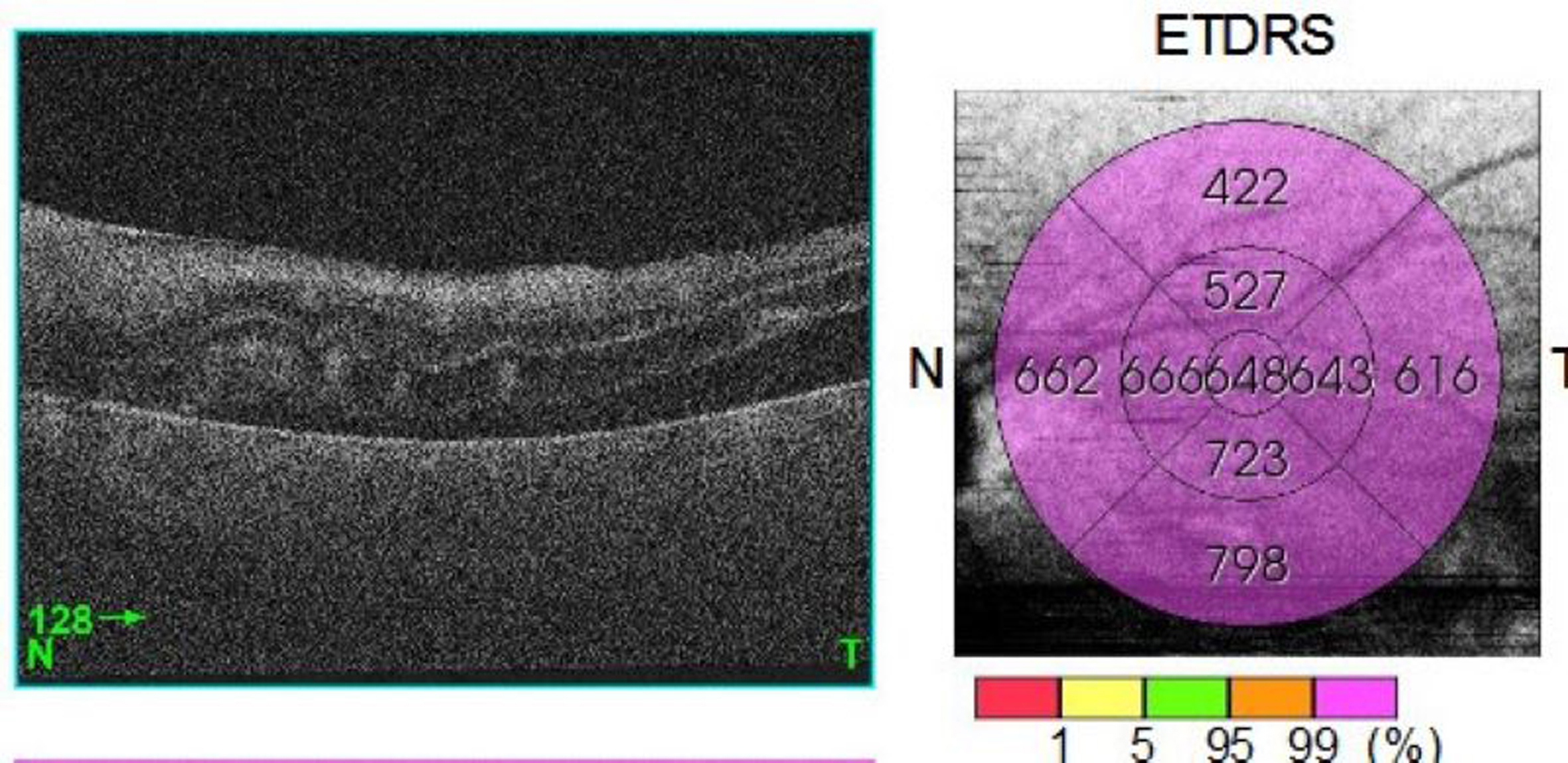

Supplement: Fig. S2 — Optical coherence tomography of the macula in Case №3, 7 days after surgery. Diffuse macular edema. [file mmcfigs2.jpg]
